# Backbone NMR assignments of the C-terminal domain of the human prion protein and its disease‐associated T183A variant

**DOI:** 10.1007/s12104-021-10005-y

**Published:** 2021-02-15

**Authors:** Máximo Sanz-Hernández, Alfonso De Simone

**Affiliations:** 1 Department of Life Sciences, Imperial College London, South Kensington, London, SW7 2AZ UK; 2 Department of Pharmacy, University of Naples “Federico II”, 80131 Naples, Italy

**Keywords:** Prion protein, Protein misfolding, Transmissible spongiform encephalopathies, Structural dynamics, Pathological T183A mutation

## Abstract

Transmissible spongiform encephalopathies (TSEs) are fatal neurodegenerative disorders associated with the misfolding and aggregation of the human prion protein (huPrP). Despite efforts into investigating the process of huPrP aggregation, the mechanisms triggering its misfolding remain elusive. A number of TSE-associated mutations of huPrP have been identified, but their role at the onset and progression of prion diseases is unclear. Here we report the NMR assignments of the C-terminal globular domain of the wild type huPrP and the pathological mutant T183A. The differences in chemical shifts between the two variants reveal conformational alterations in some structural elements of the mutant, whereas the analyses of secondary shifts and random coil index provide indications on the putative mechanisms of misfolding of T183A huPrP.

## Biological context

The misfolding and aggregation of the human prion protein (huPrP) is directly linked to the development of a group of fatal neurodegenerative disorders known as transmissible spongiform encephalopathies (TSEs), including Creutzfeldt-Jakob disease (CJD), kuru or fatal familial insomnia (FFI) (Prusiner 1998). The native huPrP denoted as the cellular form (huPrP^C^) is believed to misfold and aggregate into the disease-associated scrapie form (huPrP^Sc^). Misfolded huPrP^Sc^ can propagate its conformational state by acting as a protein-only infectious agent (Telling et al. 1996). huPrP^C^ is a glycosylated protein that is bound to the plasma membrane via a C-terminal glycosylphosphatidylinositol (GPI) anchor (Aguzzi et al. 2008). The N-terminal domain of huPrP^C^ (residues 23–124) is intrinsically disordered and contains five copper-binding octapeptide repeats, whereas the C-terminal domain (residues 125–230, here denoted huPrP^C^_125–230_) has a globular structure, composed of three α-helical segments (H1, H2 and H3) and two short β-strands (S1 and S2) forming an antiparallel β-sheet. The structure of huPrP^C^_125–­­­230_ has been experimentally determined by NMR (Zahn et al. 2000) and X-ray crystallography (Knaus et al. 2001).

In humans, TSEs may arise sporadically or can be associated with inherited mutations in the prion gene, which are primarily located in the C-terminal domain of huPrP, with nearly 40 TSE-associated single point mutations currently known (Minikel et al. 2016). Equilibrium unfolding experiments have determined that T183A is the most destabilizing TSE-associated mutation huPrP^C^ (Liemann and Glockshuber 1999), resulting in the disruption of a crucial hydrogen bond between the sidechain of T183 and backbone atoms of Y162 (De Simone et al. 2007). This mutation is linked with a phenotype of very early-onset spongiform encephalopathy (Nitrini et al. 1997). In this work, we measured of the NMR resonances of T183A huPrP^C^_125–230_ and compared their values with those of the wild type (WT) protein. The analysis of the chemical shifts indicates possible alterations of the native structure and dynamics associated with the pathological mutation. The differences between the NMR resonances of the two forms of the protein may suggest crucial insights into the mechanisms of onset of prion misfolding in the context of familial forms of TSE.

## Methods and experiments

Constructs for WT and T183A huPrP^C^_125–230_ contained an N-terminal His-tag followed by a TEV cleavage site. Transformed *E. coli* BL21 (DE3) pLysS cells were initially grown in 2xTY medium to an OD_600_ of 1.5, and then resuspended in M9 minimal medium containing 0.7 g/L ^15^NH_4_Cl and 2.0 g/L ^13^C D-glucose. Expression was induced by addition of 1 mM IPTG and cells were harvested after 3 h of expression at 37 °C. huPrP^C^_125–230_ was purified from the insoluble fraction using Ni-NTA agarose resin in a buffer containing 6 M GdHCl, 100 mM Na_2_HPO_4_, 10 mM reduced L-glutathione, pH 8.0. The purified fractions were refolded by a 1:20 dropwise dilution at 4 °C, into a 100 mM Na_2_HPO_4_, pH 7.0 buffer. Samples were then concentrated using a stirred cell, and the tag was cleaved by addition of TEV protease at a 1:10 molar ratio. Proteins were further purified by reverse nickel affinity chromatography after cleaving, followed by size exclusion on a Superdex 75 column to ensure their monomeric state.

NMR experiments were performed in ^1^H, ^13^C and ^15^N labelled samples, in a buffer containing 100 mM Na_2_HPO_4_, 10 % D_2_O, pH 7.0 at 289K. Spectral assignment of the backbone resonances was achieved using a combination of ^1^H-^15^N HSQC, HNCA, HN(CO)CA, CBCANH, CBCA(CO)NH, HNCO and HN(CA)CO spectra as previously done (Fusco et al. 2012). Experiments were performed using an 18.8 T (800 MHz ^1^H frequency) Advance III HD Bruker Spectrometer. The spectra were processed and analysed using NMRPipe (Delaglio et al. 1995) and CCPNAnalysis (Vranken et al. 2005). Chemical shift analysis was performed using the CSI 2.0 web server (Hafsa and Wishart 2014).

## Extent of assignments and data deposition

**Fig. 1:**
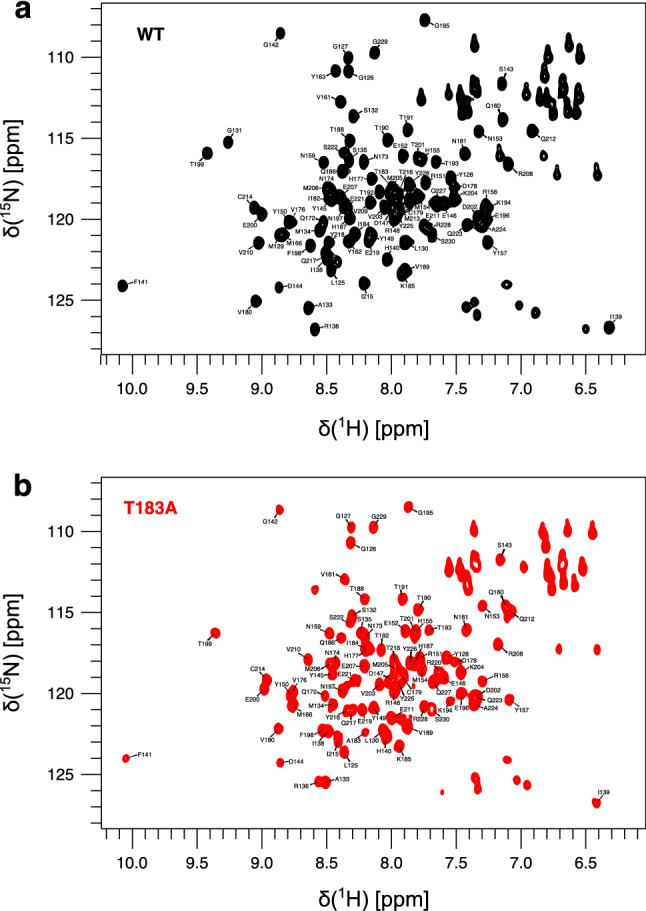
2D [^1^H-^15^N] HSQC spectra of huPrP^C^_125−230_ showing the assignment of the backbone resonances. The spectra were recorded at 289K and pH 7.0 on an 18.8 T (800 MHz ^1^H frequency) spectrometer and at protein concentrations of 100 µM. **a** WT, **b** T183A

NMR spectra of both WT and T183A variants of huPrP^C^_125−230_ were acquired at 289K and pH 7.0. Under these conditions we could assign 95 and 89 ^1^H-^15^N correlations for WT and T183A huPrP^C^_125230_, respectively, out of a possible 103 non-proline residues (Fig. 1). Both constructs showed the spectral properties typical of structured proteins as indicated by the dispersion of resonances in the ^1^H-^15^N HSQC spectra. In particular, the spectrum of WT huPrP^C^_125–230_ is consistent with previously published data, suggesting that the protein is in the native state under these experimental conditions (Zahn et al. 2000). Samples of the WT huPrP^C^_125–230_ could be concentrated above 300 µM without loss of NMR signal over time or visible precipitation in the NMR tube. By contrast, T183A huPrP^C^_125–230_ showed some level of instability, with prompt precipitation at concentrations higher than 100 µM. Moreover, at 100 µM 32 % of the signal was found to be lost after 18 hours of incubation at 289K. These experimental conditions allowed for 3D NMR spectra for backbone assignment to be measured despite the inherent instability of the mutant protein, but at the cost of a drastic reduction in the signal to noise ratio. The signal reduction was significant for CBCANH and CBCA(CO)NH spectra, thereby reducing the amount of assigned ^13^C_β_ resonances with respect to WT huPrP^C^_125–230_ (Table 1).

**Table 1:** Assignment coverage in WT and T183A huPrP^C^_125–230_

	Number of assigned nuclei				
	^13^C_α_	^13^C_β_	^13^C_O_	^15^N	^1^H_N_
WT	96	76	94	95	95
T183A	93	13	89	89	89

Out of 103 non-proline residues

Resonances missing from the assigned spectra of WT huPrP^C^_125–230_ mostly correspond to the loop connecting the β-strand S2 and the α-helix H2, spanning residues 164 to 171, whose signals are expected to broaden due to conformational exchange (Damberger et al. 2011). In addition, other missing resonances were associated with residues F175 and R220. In the case of T183A WT huPrP^C^_125–230_, several more residues were found to lack NMR peaks in the spectra. The missing S2-H2 region extended from residues 162 to 171. Other undetected resonances included residues M129, G131, F175, I182, V209 and M213. The spectral properties of T183A huPrP^C^_125–230_ therefore suggested enhanced conformational exchange in this protein construct.

**Fig. 2:**
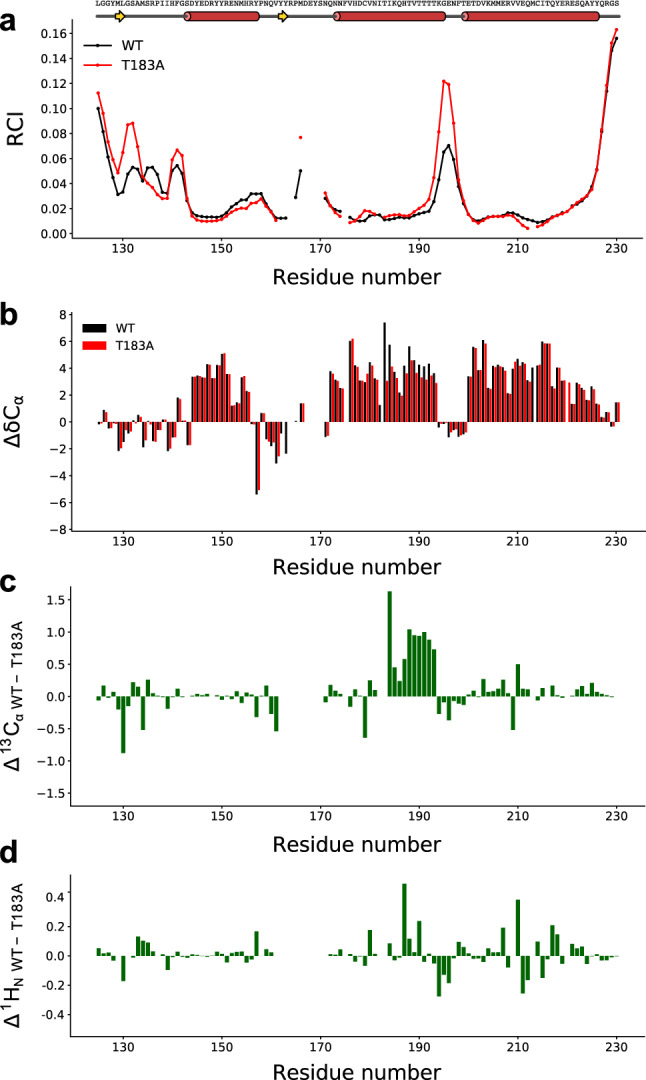
**a** Random coil index (Berjanskii and Wishart 2005) along the sequence of WT (black) and T183A (red) hPrP^C^_125–230_. The sequence and secondary structure schematic are displayed on the top. **b** Secondary ^13^C_α_ chemical shifts for WT (black) and T183 (red) constructs. **c**, **d** Raw ^13^C_α_ (**c**) and ^1^HN (**d**) chemical shift differences between WT and T183A hPrP^C^_125–230_

We analysed the chemical shift values to calculate the random coil index (RCI) (Berjanskii and Wishart 2005). This analysis generated RCI profiles that are generally in line with the structural elements of huPrP^C^_125–230_ (Fig. 2a), suggesting that the native fold is preserved in both WT and T183A. However, in the loop regions of T183A huPrP^C^_125–230_, we found a sharp increase in the RCI values, indicating an enhancement of the flexibility and structural dynamics as a result of the pathological mutation. We also evaluated the secondary ^13^C_α_ chemical shifts of both variants, which are sensitive probes of secondary structure elements in proteins (Wishart and Sykes 1994). Secondary ^13^C_α_ chemical shifts were calculated as the experimental chemical shifts minus the random coil values (calculated using the PROSECCO server, Sanz-Hernandez and De Simone 2017) , resulting in similar profiles across large portions of the PrP sequence in the two variants. Some differences, however, were observed in the C-terminal region of the α-helix H2, with reduced secondary shifts in the T183A variant with respect to the WT protein, suggesting a lower content of α-helix. The destabilisation of the α-helix H2 ranges residues 184–194, suggesting that the mutation of T183 has downstream effects of structural destabilisation into the sequence. By contrast, no significant alterations of secondary chemical shifts were found in correspondence of the α-helix H1 (residue 144–155), which is prone to misfolding once detached from the native environment (Camilloni et al. 2011). The difference in secondary shifts between the two variants are clearer when plotting the raw differences of between ^13^Cα and ^1^HN chemical shifts (Fig. 2c, d) showing a localised perturbation in the region 184–194 of the protein.

In summary, the NMR assignments of WT and T183A huPrP^C^_125–230_ under native conditions reveal noticeable differences in the conformational properties of the protein, featuring enhanced structural dynamics in the case of the pathological mutant. The availability of these assignments will allow further studies at high-resolution characterisation of the misfolding mechanisms in this familial form of TSE.

## Data Availability

The assignments have been deposited to the BMRB under the accession codes: 50,527 (WT) and 50,528 (T183A).
